# Home office versus ergonomic workstation - is the ergonomic risk increased when working at the dining table? An inertial motion capture based pilot study

**DOI:** 10.1186/s12891-022-05704-z

**Published:** 2022-08-03

**Authors:** Fabian Holzgreve, Christian Maurer-Grubinger, Laura Fraeulin, Juliane Bausch, David A. Groneberg, Daniela Ohlendorf

**Affiliations:** 1 Institute of Occupational Medicine, Social Medicine and Environmental Medicine, Goethe- University Frankfurt, Theodor-Stern-Kai 7, Building 9a, 60596 Frankfurt am Main, Germany; 2 Institute of Sport Sciences, Goethe-University Frankfurt, Frankfurt am Main, Germany

**Keywords:** Ergonomics, Ergonomic risk potential, Kinematic analysis, Xsens, Inertial motion units, Rapid upper limb assessment, RULA

## Abstract

**Background:**

In order to reduce the risk of infection with Sars-Cov-2, work practices have been shifted to the home office in many industries. The first surveys concerning this shift indicate an increase in musculoskeletal complaints of many employees. The aim of this study was to compare the ergonomic risk in the upper extremities and trunk of working in a home office with that of working in an ergonomically optimized workplace.

**Methods:**

For this purpose, 20 subjects (13w/7m) aged 18–31 years each performed a 20-minute workplace simulation (10 min writing a text, 10 min editing a questionnaire) in the following set up: on a dining table with dining chair and laptop (home office) and on an ergonomically adjusted workstation (ergonomically optimized workplace). The subjects were investigated using a combined application of a motion capture kinematic analysis and the rapid upper limb assessment (RULA) in order to identify differences in the ergonomic risk.

**Results:**

Significantly reduced risk values for both shoulders (left: *p* < 0.001; right: *p* = 0.02) were found for the ergonomically optimized workstations. In contrast, the left wrist (*p* = 0.025) showed a significantly reduced ergonomic risk value for the home office workstation.

**Conclusion:**

This study is the first study to compare the ergonomic risk between an ergonomically optimized workplace and a home office workstation. The results indicate minor differences in the upper extremities in favor of the ergonomically optimized workstation. Since work-related musculoskeletal complaints of the upper extremities are common among office workers, the use of an ergonomically optimized workstation for home use is recommended based on the results.

## Background

In the wake of the SARS-CoV-2 pandemic, employees worldwide shifted their workplaces from the office to their homes, in order to reduce the risk of infection and to protect their health. However, this was accompanied with the fact that the equipment of home offices may not meet the same ergonomic standards as those of the office workplace [1]. Previously, only 4% of workers regularly worked in a home office, however, during the first lockdown, this proportion increased to 27% in Germany [2]. Even before the pandemic, only 7% of home office workers had ergonomic work equipment, according to Janneck et al. [3]. Thus, many employees are now forced to perform their work at the kitchen table or on the couch, amongst other places. Furthermore, the laptop is usually not aligned according to ergonomic guidelines [4], but is simply placed on the table top or on the lap. Likewise, the screens of mobile devices are generally many times smaller than ergonomic monitors. According to the German Social Accident Insurance (DGUV) [5], the distance between the eyes and the screen should ideally be 50–70 cm, while attention should also be paid to suitable light incidence. In addition, the mouse and keyboard should be external, i.e., separate from the screen, to support an ergonomic posture [6]. The kitchen chair is also usually not designed to be used for several hours at a time; for example, it has no supportive section in the lumbar region and often no armrests. In addition, not many dining tables possess a height-adjustable and, thus, ergonomic table top. In a survey conducted by Forsa in the spring of 2021, 36% of respondents stated that back pain or headaches had occurred due to poor ergonomic workplace equipment. In this context, 34% criticized the lack of, or inadequate, workplace equipment at home [7]. According to Gerding et al. [8], over 40% of home office workers reported suffering from moderate to severe pain, with affected regions primarily representing the lower and upper back, neck and eyes. Moretti et al. [9] also found an exacerbation of neck pain in 50% of home office workers. In contrast, Argus and Pääsuke [10] could not detect significant differences in the prevalence of musculoskeletal complaints before and during the lockdown in Estonia. Aegerter et al. [11], in turn, indicated strong evidence for poorer ergonomic conditions in the home office compared with the conditions in the office [11]. The aforementioned studies deal almost exclusively with survey research. Thus, observational methods or inertial motion capture systems could complete these statements with quantitative data.

**Fig. 1:**
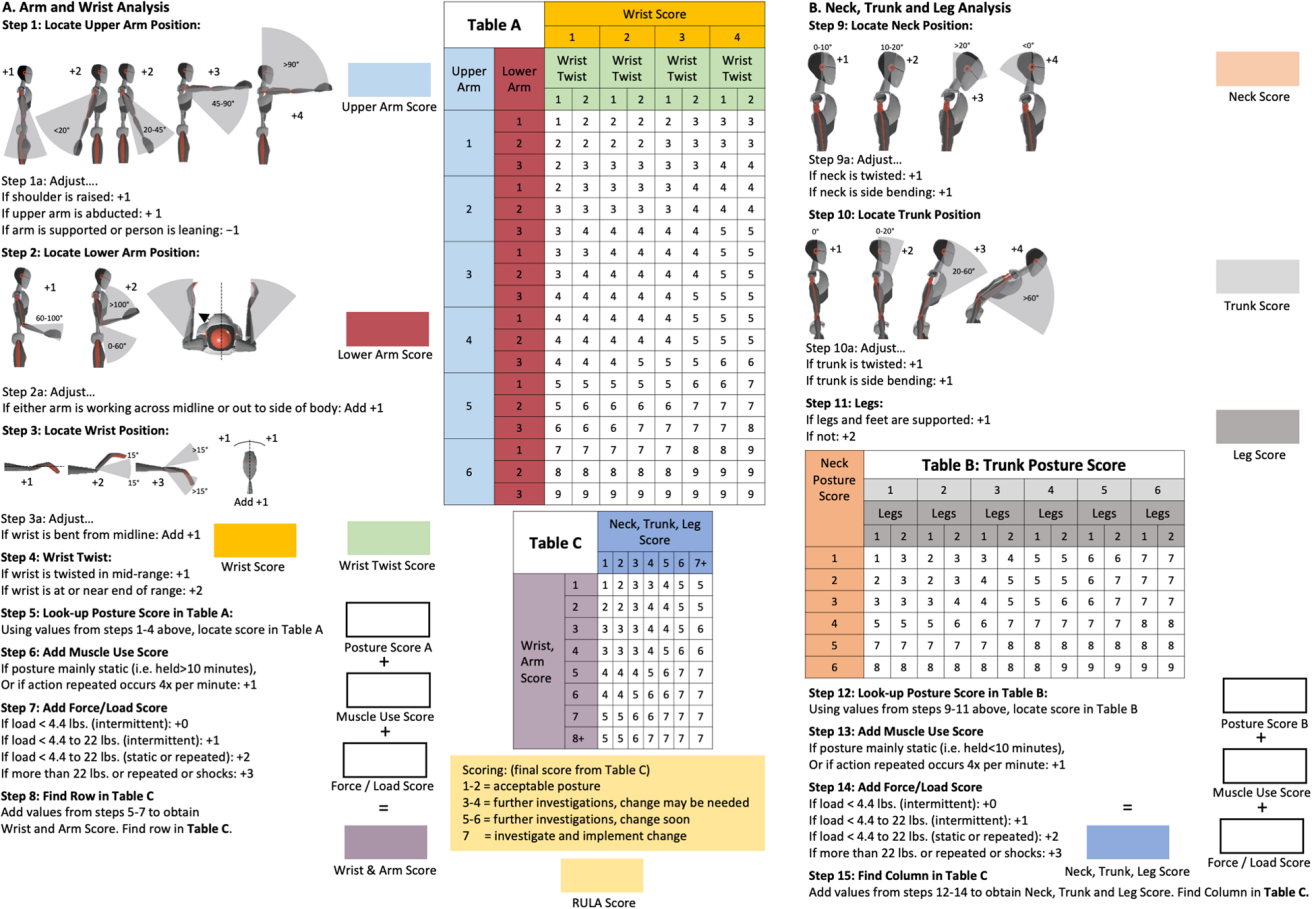
The ‟Rapid Upper Limb Assessment (RULA)” worksheet [14]

The Rapid Upper Limb Assessment (RULA) is an example of an observational method for the ergonomic risk assessment of workplaces. Here, an overall value is calculated by means of a worksheet (Fig. 1), which quantifies the load on the entire body and also on individual body parts [12]. This can then be used to draw conclusions about the hazard potential, although it should be noted that a concrete relationship between the RULA total value and the actual risk potential has not yet been adequately demonstrated [13]. The major disadvantage of this observational method is that complete objectivity is not guaranteed since the observers objectify the posture subjectively. For example, projections when the camera is not positioned orthogonally to measured joint angles, joints that are difficult to see, or simply the challenge of evaluating 2–3 degrees of freedom of a joint by eye can make optimal evaluation difficult. Furthermore, the RULA total value is only the evaluation of a snapshot and, thus, is basically more suitable for static positions.

A combination of motion analysis and RULA offers the possibility to quantify the ergonomic classification of postures and to evaluate the whole motion sequence at the same time. Such a method was developed using the motion analysis method of Inertial-Motion-Capture [14] and this has already been applied in different investigations [15–17]. Inertial motion units have the advantage of being relatively easy to deploy in the field, making them suitable for studies of different groups of workers directly in the workplace.

The aim of the present study was to determine experimentally, and to compare, the ergonomic risk of a home office set up and an ergonomically designed workplace by means of an inertial measurement unit (IMU) analysis [13] combined with RULA. In this context, priority was given to those parts of the body that are exposed to a particular risk.

## Materials & methods

### Subjects

A total of 20 subjects (13w/7m) between the ages of 18–31 years participated voluntarily in this pilot study. The subjects were sports students of the Goethe University Frankfurt am Main (Germany); their subject data are presented in Table 1. A minimum age of 18 years was required for the subjects to be included whilst exclusion criteria comprised on the basis of self-reporting the use of perception-altering substances, acute injuries or severe diseases (cardiovascular/pulmonary/renal dysfunction, neurological/psychological diseases, advanced degenerative diseases of the musculoskeletal system and not fully healed injuries affecting the quality of life or physical performance.

**Table 1:** Summary of the subjects’ data

	Age [years]	Height [cm]	Weight [kg]	BMI [kg/m^2^]	Sports/week [hours]	Hand dominance [%]
Mean^1^ / Median^2^	23.50^2^	163.75^2^	56.50^2^	23.19^1^	6.94^1^	90% right
Standard deviation^1^ / Interquartile distance^2^	5.75^2^	15.25^2^	14.75^2^	2.78^1^	3.47^1^	10% left

Symbol superscripts ^1^ and ^2^ indicate whether data are normally distributed (mean, standard deviation) or not normally distributed (median, interquartil distance)

 All subjects gave written informed consent in advance to participate in the study. The study was approved by the Ethics Committee of the Department 05 Psychology and Sports Science of the Goethe University Frankfurt (2020-59).

### Workplace conditions and tasks

In order to investigate the ergonomic hazards of working in a home office, it was necessary to define a representative workplace arrangement for working in a home office. The “home office” (HO) workstation arrangement consisted of a typical dining table with a height of 72 cm and a 47 cm high dining chair. A 15.6-inch laptop and mouse were used for task processing. The position of the laptop and the mouse, as well as the tilt angle of the screen, could be set individually. The workplace arrangement “ergonomic workplace” (ERGO) was based on the ergonomic recommendations of the German Social Accident Insurance (DGUV) [18]. Accordingly, a height-adjustable desk, a desk chair and a height-adjustable monitor were part of the workplace arrangement.

Two different tasks were chosen to represent office work. Firstly, a digital questionnaire was filled in using a mouse and keyboard. This consisted of 2 well-validated questionnaires; the Nordic Questionnaire [12] assessing musculoskeletal complaints and the SF-36 [19] assessing health-related quality of life. On the other hand, the test persons had to copy out a given text with all its details. Subjects were asked to complete each task for 10 min. If the questionnaire was completed beforehand, it was repeated.

### Inertial Motion capture System

For all kinematic recordings the inertial motion capture system MVN Link from Xsens (Xsens Technologies B.V., Enschede, The Netherlands) was used. For this purpose, 17 inertial sensors, a minicomputer (body pack) and a battery were attached to a special suit. Each sensor contains a linear 3D accelerometer, a gyroscope, a barometer and a magnetometer that internally samples at 1000 Hz [20]. According to the manufacturer, the sampling rate of the total output is 240 Hz and the measurement error is specified as ± 1%. Compared to optical motion capture (gold standard), this inertial motion capture system provides good to excellent data in terms of simultaneous findings, especially in the frontal and sagittal planes [21, 22]. The Xsens system interpolates, among others, a total of 22 joints with 3 dimensions and data on the position and orientation of 23 segments. All recordings were performed using the “No Level” scenario, a mode in which the limbs and segments are viewed relative to the pelvis. The “No Level” scenario is included in the Xsens Analyze software and provides the best data quality for ergonomic analysis.

### Rapid Upper Limb Assessment (RULA)

In order for a risk assessment to be carried out using kinematic data, such data must be backed up by an ergonomic assessment matrix.

RULA is used to assess the ergonomic risk of work processes. The procedure focuses on individual body regions such as the neck, shoulders, upper body, arms and hands. Using a series of images of different postures, an overall “global” posture can be quantified (Fig. 1) [23–25]. Here, the assessment protocol is divided into three main steps. Step A includes measurements of the upper and lower arms and wrists. Step B includes the measurements of the neck, trunk and legs. In step C, the total value is calculated from which ergonomic recommendations are derived (Fig. 1) [14, 23, 25].

### Data processing

The recorded data were first processed in the MVN Analyze software provided by Xsens; all data were checked for errors (e.g. magnetic disturbances, drift errors, errors in the execution of the task), extracted and assembled into mat. files. The RULA coding system was slightly modified in some parts by rewriting it into a Matlab code. This modification was necessary because not all steps were suitable for application to objective kinematic data. The Matlab code, the necessary modifications of the RULA coding system and the explanations in detail are published in a specific method article in Maurer-Grubinger et al. [14].

In the current study, data have been analyzed in a drop-down procedure with several levels of complexity. For the most global approach, the median of the RULA total score was used. In addition, since the entire work processes were recorded over several minutes, it was possible to determine how much time the subjects spent, in relative terms, in each RULA score (score 1–7) and, thus, the relative time values could be obtained. This provides a more accurate view of the ergonomic hazard of each body part for the right or left side of the body. At this level, we included Step 1 (upper arm score), Step 2 (lower arm score) and a combination of Steps 3 and 4 (wrist score) proposed by Vignais et al. [26], as well as Step 9 (neck score) and Step 10 (trunk score).

In summary, the combination of the RULA and kinematic data allows a differentiated assessment of the ergonomic risk based on two outcome variables:

1. Median + interquartile range.
2. Relative time value.

The relative time value was calculated as follows:

relative time score of RULA score 1*1 + relative time score of RULA score 2*2 + relative time score of RULA score 3*3(.) + relative time score of RULA score 7*7.

### Measurement protocol

The measurements on the subjects were carried out in small groups on different days and at different times. In the first place, the test persons received an introduction to the measurement procedure and the execution process. Subsequently, the order of the conditions (ergonomic vs. home office workplace and “filling out questionnaires” vs. “writing text”) was randomized. The measurements always took place on two subjects at the same time. The ergonomic workplace was previously adapted to the respective participant according to the specifications of the DGUV [18]; this included, for example, adjustments to the seat height, armrest height, screen distance, etc. The screen (either laptop or monitor) was positioned orthogonal to the windows, which were slightly dimmed. The measurements took place during the day at noon, in order to enable similar environmental conditions. Each task was performed in both workstation arrangements for a respective duration of 10 min. The pure measurement duration was, therefore, 40 min per subject and 20 min per workstation.

### Statistical analysis

For the statistical analysis of the subject and kinematic data, the Lilliefors test was used to test for normal distribution. Since the majority of the data were not normally distributed, non-parametric procedures were chosen. Thus, for the descriptive statistical analysis of the subject data, the median and interquartile range (except for the parameter of sports/week) were calculated. For the inferential statistics of the group comparisons in the context of risk assessment using RULA, the Wilcoxon signed-rank test was applied. Microsoft Excel 2016 (Microsoft Corporation, Redmond, WA, USA), Matlab R2020a (The Mathworks Inc., Natick, MA, USA) and IBM SPSS v28 (International Business Machines Corporation (IBM), Armonk, NY, USA) were used for statistical analyses. The significance level was set at α = 5%.

## Results

### Descriptive statistics

**Fig. 2:**
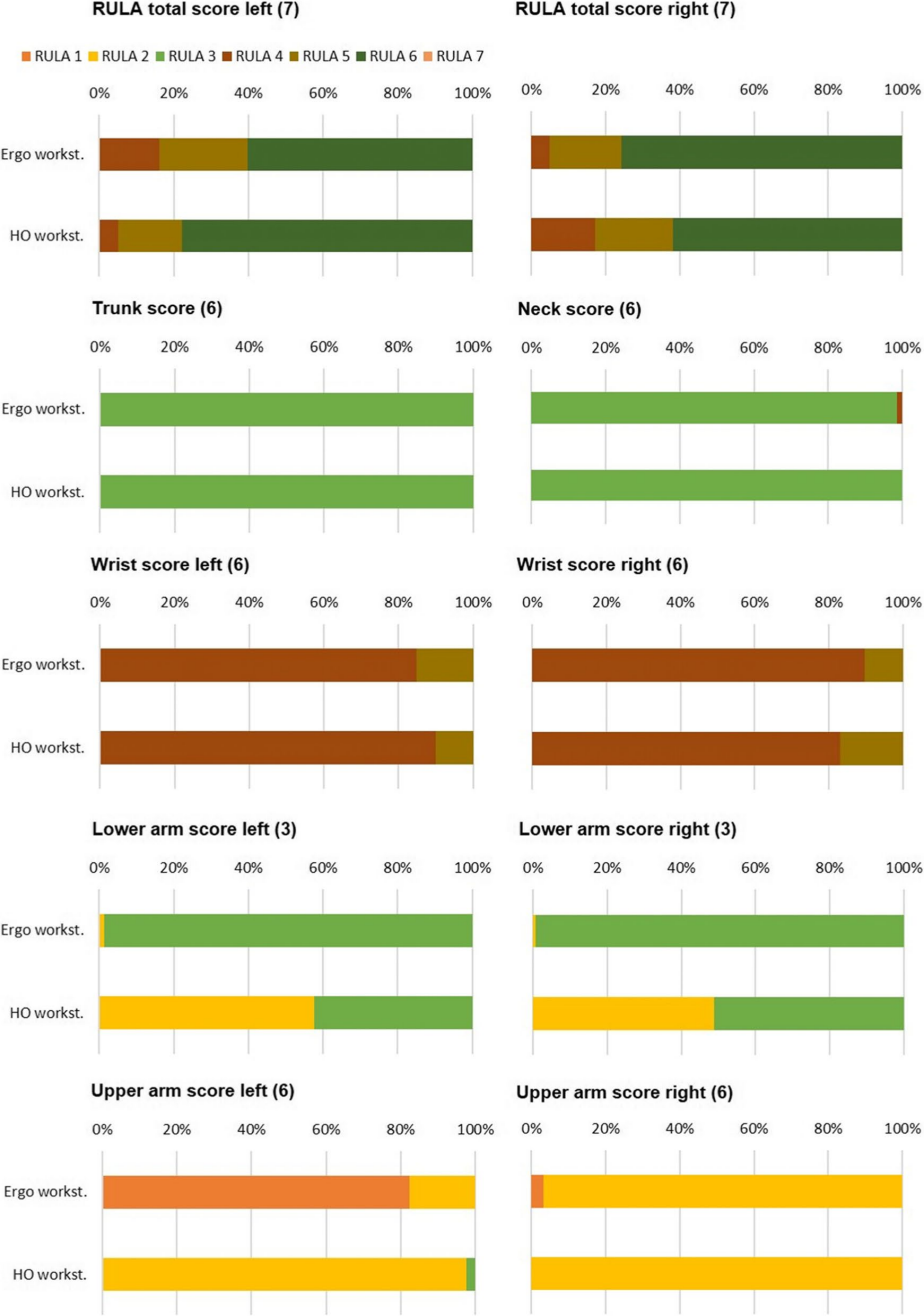
Relative distribution of the respective RULA scores by body half and region for the ergonomic (ERGO) and home office (HO) workplace arrangement. The maximum achievable RULA score is shown in parentheses behind the regions

In the RULA total score of the descriptive analysis of the relative time distributions, small differences between the conditions of the ergonomic- and home office workplaces could be seen, as well as differences between the left and right halves of the body (Fig. 2; Table 2). While for the left RULA, the total score in the ERGO revealed that about 60% of the time was spent in RULA 6, the value for RULA 6 in the HO was almost 80%. Accordingly, the time shares for RULA 4 & 5 in the ERGO were also lower; this pattern was also reflected in the medians (Table 2). In contrast, the RULA total score for the right half of the body was almost the opposite. Here, the subjects in the ERGO worked almost 80% of the time in RULA 6 whilst the proportions of RULA 4 were marginal; the medians (median and relative time score) also indicated this trend (Table 2). Accordingly, the risk assessment for the HO was better, with only 60%, or slightly more of the time being spent in RULA 6 and almost 20% in RULA 4.

No differences were observed in the trunk and neck score. Here, the subjects were in RULA 3 for almost the entire time in both conditions examined. Analogously, the medians also indicated a RULA value of 3 (Table 2).

The wrist score clearly indicated a lower ergonomic risk for the left half of the body in a side-by-side comparison (Table 2). Furthermore, a higher ergonomic risk in the left half of the body was indicated for the ERGO with about 90% of the time spent in RULA 4 and 10% in RULA 5 when compared to the HO which, likewise, exhibited a correspondingly higher proportion in RULA 4 and a lower proportion in RULA 5 (Fig. 2); this was also reflected in the medians (Table 2). The right half of the body paints a correspondingly reversed picture with a slightly higher ergonomic risk for the HO (Fig. 2; Table 2).

In the lower arm score, the side differences were negligibly small, however, differences between the working conditions were more evident here. While the subjects worked in RULA 3 for almost the entire time in the ERGO, the proportion of RULA 2 in the HO was almost 50% (Fig. 2). The same pattern was exhibited by the medians with increased RULA values for both halves of the body, which is shown in Table 2.

In contrast, the left upper arm score for the ERGO showed that about 80% of the time was spent in RULA 1, whereas in the HO this was 0%, with small proportions being spent in RULA 3. This pattern was also expressed in the medians, with a difference of one RULA score (Table 1). Corresponding differences were not observed for the right half of the body.

**Table 2:** Median and relative time score as well as the respective interquartile distance (IQD) of the ergonomic and home office workplace conditions for all RULA regions investigated

	Ergonomic workstation		Home office workstation	
	Median (IQD)	Relative time score (IQD)	Median (IQD)	Relative time score (IQD)
Total score right	5.5 (1)	5.45 (0.97)	5.5 (1)	5.28 (0.72)
Total score left	5 (1.5)	5.08 (1.11)	6 (1)	5.63 (0.65)
Trunk score	3 (0)	3.00 (0.00)	3 (0)	3.00 (0.00)
Neck score	3 (0)	3.02 (0.13)	3 (0)	3.00 (0.08)
Wrist score right	4 (0)	4.09 (0.41)	4 (0)	4.22 (0.22)
Wrist score left	4 (1)	4.14 (0.42)	4 (0)	4.00 (0.56)
Lower arm score right	3 (1)	2.99 (0.78)	2.5 (1)	2.51 (0.50)
Lower arm score left	3 (1)	2.99 (0.78)	2.5 (1)	2.42 (0.72)
Upper arm score right	2 (1)	1.99 (0.68)	2 (0)	2.00 (0.05)
Upper arm score left	1 (1)	1.22 (0.90)	2 (0)	2.00 (0.47)

### Inferential statistics

**Fig. 3:**
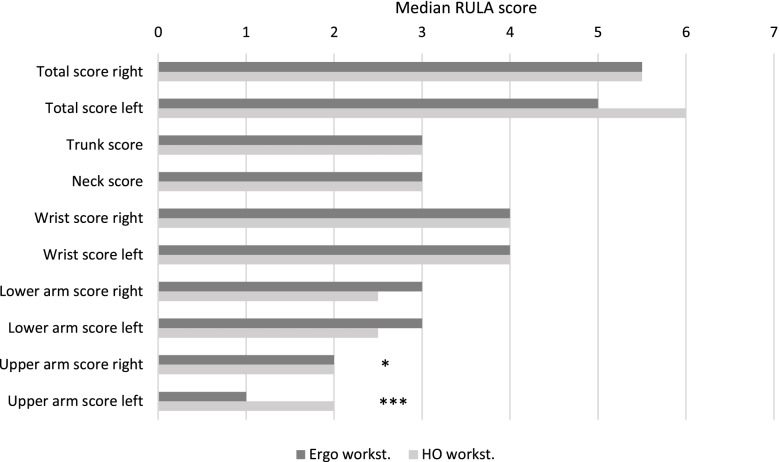
Median RULA total score per body region for the ergonomic and home office workplace conditions. Significant differences are marked with an asterisk: * = *p* < 0.05; ** = *p* < 0.01; *** = *p* < 0.001

**Fig. 4:**
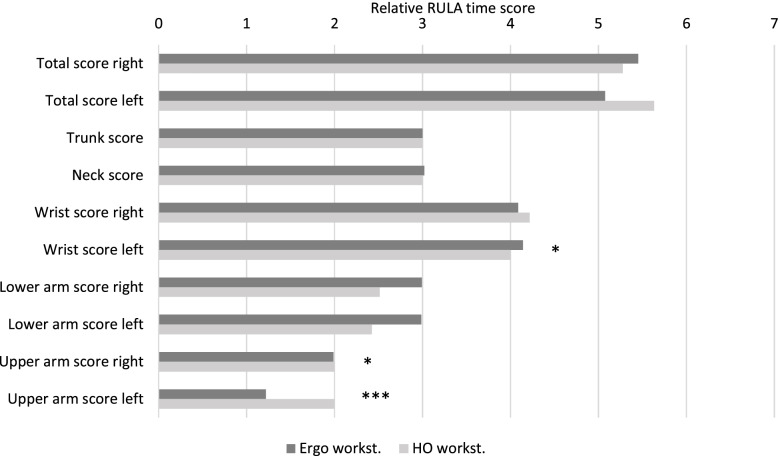
Median relative time score per body region for the ergonomic and home office workplace conditions. Significant differences are marked with an asterisk: * = *p* < 0.05; ** = *p* < 0.01; *** = *p* < 0.001

The results of the inferential statistics of both the median and relative time score show no significant results in the RULA total score (Figs. 3 and 4). However, the ergonomic risk of the left upper arm score in the ERGO was highly significantly reduced compared to that in the HO (median: *p* < 0.001; relative time value: *p* > 0.001). A significantly reduced risk could also be determined for the right upper arm score in both outcome variables in the ERGO (median: *p* = 0.02; relative time score: *p* = 0.02). Another statistically relevant difference could be observed in the relative time score for the left wrist score. The ergonomic risk of the HO was significantly lower (*p* = 0.025) than in the ERGO.

## Discussion

The purpose of this study was to quantitatively assess and compare the ergonomic risks of a non-ergonomic home office and an ergonomically designed workstation. The results of the inferential statistics suggest that the differences in the total score were small, however, there was a significantly reduced ergonomic risk observed for both shoulders (left upper arm score: *p* < 0.001; right: *p* = 0.019) when working in the ergonomic workplace (Figs. 3 and 4). In contrast, the left wrist score indicated significantly lower ergonomic risk (*p* = 0.024) for the HO workplace (Fig. 4). Looking at the results in a side-by-side comparison, the left-side dominated differences in the wrist and shoulder area could be explained by the different arrangement in the left work area between the two conditions. While in HO the keyboard is integrated in the laptop, in ERGO users can rely on an external keyboard. In contrast, the workspace of the right arm (the operation of the mouse) seems to be basically very similar for both conditions. An explanation of the results by the distribution of handedness among the subjects is not to be expected, but cannot be excluded in principle as a confounding variable. Descriptively, the results indicate a reduced ergonomic risk for the left half of the body for the ERGO compared to the HO (median: 5–6, relative time score: 5.08–5.63) in the total score (Table 2). In contrast, non-significant trends were evident in the forearms for both sides of the body, identifying an increased ergonomic risk for the ERGO over the HO (Table 2). Due to this study being, in our opinion, the first to compare the ergonomic risks of a non-ergonomic home office and an ergonomically set up workstation, the results of the inferential statistics cannot be directly compared with the results of other authors. However, the ergonomic risk of descriptive statistics can be placed in the context of some publications [27–30]. At this point, it should be noted that in these publications the RULA was determined based on trained reviewers’ observations, while the authors of the present study calculated RULA based on quantitative biomechanical analyses [14].

The present data were compared with those of other studies that used RULA according to conventional procedures. For working at an office workplace, Rodrigues et al. [29] showed similar results with mean values (confidence intervals) in a RULA total score of 5.41 (4.78–6.04) for subjects without and 5.59 (4.91–6.27) for subjects with musculoskeletal complaints. Slightly lower RULA total scores compared to the current study were shown by Bazazan et al. [27] for both study groups studied prior to implementation of a workplace intervention, with mean (SD) scores of 5.1 (0.74) for the intervention group and 5.0 (0.63) for the control group. Similar results were published by Govil et al. [28] with RULA total scores of between 3 and 6. Significantly lower RULA total scores for sedentary office work were recorded by the research group led by Taieb-Maimon [30] with mean values of 4.02 (0.52). Nevertheless, the magnitude of the results in the RULA total scores of the current study seems to be consistent with the results of published studies for sedentary office work [27–30]. Rodrigues et al. [29] found that especially the seat height, armrest and backrest increase the risk potential of a non-ergonomic workplace [29], while the results of the current study show an increased risk potential for the shoulder and the upper arm (Figs. 3 and 4). Especially here, the ergonomic workplace adjustments in terms of seat and desk height adjustment, as well as armrest adjustment, seem to reduce the ergonomic risk in the shoulder region. This is of great value since work-related neck pain is associated with unergonomic shoulder/scapula postures [31], thus, as neck pain is highly prevalent in office workers, such findings are of clinical relevance.

In contrast, these workstation adjustments appear to have less effect on the posture in the trunk region (Figs. 3 and 4). However, current studies show that the neck and trunk regions, especially, are the body regions most affected by work-related musculoskeletal complaints for sedentary office work [32, 33]. According to the results of the current study, ergonomic workplace alignment does not lead to reduced ergonomic risk for these body regions. The significance of the presumably relevant difference in posture between the conditions (height-adjustable desk and chair versus fixed desk and chair heights) may have been significantly reduced by the large proportion of female subjects and the associated low median body height; this would result in reduced differences in posture and, thus, ergonomic risk between the conditions studied in particular. Since RULA assesses the postural deviations in the neck and trunk in the frontal, sagittal and transverse planes (Fig. 1), only deviations due to movements in the sagittal plane would be expected due to the task in the context of this study. In particular, for small body sizes, the assessment by RULA in the neck and trunk regions may not be sufficiently sensitive to the main characteristics of the studied conditions as smaller individuals benefit from the average chair and table heights used. Nevertheless, although the results suggest a reduced relevance for the effect of ergonomic workplace design for work-related musculoskeletal complaints in the neck and trunk regions, the results for the neck and trunk do not seem to be fully justified.

This pilot study provides precise information on the ergonomic strain and enables quantitative conclusions about the hazard potential of a non-ergonomic workplace. Evidence of increased ergonomic strain, particularly in the upper extremity, was shown in students when working at home compared to ergonomically optimized workstations. From this arises the need to compare in future follow-up studies the ergonomic risk of home office workstation arrangements with those of employees in occupational groups in the service sector, since this economic sector employs the largest proportion of employees affected by home office. Important insights for this are provided by this study regarding the measurement duration at the workplace against the background of muscular fatigue and the need to investigate different home office settings. Further methodological advantages can be achieved by using gloves specially designed for measuring the kinematics of finger movement against the background of occupational diseases such as carpal tunnel syndrome.

In future studies, the importance of relevant differences in body posture in the sagittal plane should be increasingly considered in the study design. Another interesting point to be explored in future studies is the influence of font size and gaze distance on posture.

## Limitations

- The measurement duration was set to 20 min, since in our opinion this relates to a typical concentration segment during work. This leads to a total measurement time of 40 min. Given the time needed for calibration and putting on and off the suit, it took more than one hour to measure each subject. However, a typical work day lasts much longer. The comparatively short measurement duration per condition of 20 min probably did not lead to local muscular fatigue. A measurement duration based on the duration of a typical working day of office workers would have achieved such fatigue symptoms. It is expected that ergonomically-adjusted workstations would compensate better for this, so that differences in posture may be more readily identified. Possible fatigue effects could influence posture throughout the day, but this possible effect is disproportionate to the strain on subjects to wear an extremely tight suit for such a long time and time resources. It should be noted that this is a pilot study.
- The measurement period may be too short to compensate for the direct influence of habitual position changes.
- The standard for setting up the workstation at home was the same as in the office, but, as this could not be adhered to routinely due to a lack of conditions at home, the workstation design of a representative home office workstation in this study was based on work at the dining room table using an ordinary chair. When classifying the results, it must be taken into account that there is no “one” workplace arrangement at home.
- The rather small sample size is due to the fact that this is a pilot study. However, the sample size is sufficient in order to show significant differences especially in the upper extremity.

## Conclusion

In this investigation the ergonomic risk between an ergonomically optimized workplace and a home office workstation was compared by means of motion capture analysis. Minor ergonomic advantages in the ergonomically optimized workstation over the home office workstation were revealed. These are located in particular in the upper extremity. Many office workers are affected from work-related musculoskeletal complaints of the upper extremity which can lead to work disability. The results from this study based on a motion capture analysis indicate the use of an ergonomically optimized workstation for office work at home.

## Data Availability

All data generated during this study are included in this published article.

## References

[1] Herrmann, Mario, and Regina Frey Cordes. “Homeoffice Im Zeichen Der Pandemie: Neue Perspektiven Für Wissenschaft Und Praxis?“: IUBH Discussion Papers-Human Resources, 2020.

[2] Ahlers, Elke, Sandra Mierich, and Aline Zucco. “Homeoffice in Zeiten Von Corona Risiken Abwenden-Potenziale Nutzen.“ Düsseldorf (WSI Report, *65*) (2021).

[3] Janneck Monique, Jent Sophie, Helge Nissen (2018). "Ergonomics to Go: Designing the Mobile Workspace. Int J Human–Comput Interaction.

[4] Ducki, Antje. “Digitale Transformationen–Von Gesundheitsschädigenden Effekten Zur Gesundheitsförderlichen Gestaltung.“ In Fehlzeiten-Report 2019, 1–13: Springer, 2019.

[5] (DGUV), Deutsche Gesetzliche Unfallversicherung e.V. “Home-Office: So Bleibt Die Arbeit Sicher Und Gesund.“ (Accessed 22.10.2021).

[6] Schlick Christopher (2018). Ralph Bruder, and Holger Luczak.

[7] DEKRA/forsa. “Dekra Arbeitssicherheitsreport 2021 Belastung · Gesundheit · Digitalisierung. Eine Dekra/Forsa Befragung in Mittelständischen Unternehmen.“ 2021.

[8] Gerding, Thomas, Megan Syck, Denise Daniel, Jennifer Naylor, Susan E Kotowski, Gordon L Gillespie, Andrew M Freeman, Thomas R Huston, and Kermit G Davis. “An Assessment of Ergonomic Issues in the Home Offices of University Employees Sent Home Due to the Covid-19 Pandemic.“ Work, no. Preprint (2021): 1–12. 10.3233/WOR-205294 33867366

[9] Moretti Antimo, Menna Fabrizio, Aulicino Milena, Paoletta Marco, Liguori Sara, Iolascon Giovanni (2020). "Characterization of Home Working Population During Covid-19 Emergency: A Cross-Sectional Analysis. Int J Environ Res Public Health.

[10] Argus, Martin, and Mati Pääsuke. “Effects of the Covid-19 Lockdown on Musculoskeletal Pain, Physical Activity, and Work Environment in Estonian Office Workers Transitioning to Working from Home.“ Work, no. Preprint (2021): 1–9. 10.3233/WOR-210033 34180447

[11] Aegerter, Andrea M, Manja Deforth, Venerina Johnston, Gisela Sjøgaard, Thomas Volken, Hannu Luomajoki, Julia Dratva, Holger Dressel, Oliver Distler, and Achim Elfering. “No Evidence for an Effect of Working from Home on Neck Pain and Neck Disability among Swiss Office Workers: Short-Term Impact of Covid-19.“ European Spine J. 2021;1–9. 10.1007/s00586-021-06829-w PMC8019586 33817763

[12] Kuorinka I, Jonsson B, Kilbom A, Vinterberg H, Biering-Sorensen F, Andersson G, Jorgensen K (1987). Standardised Nordic Questionnaires for the Analysis of Musculoskeletal Symptoms. Appl Ergon..

[13] Maltry L, Holzgreve F, Maurer C, Wanke EM, Ohlendorf D. Präzisere Ergonomische Risikobeurteilung Durch Die Kombination Von Inertialsensoren Mit Observatorischen Methoden Am Beispiel Von Rula. Zentralblatt für Arbeitsmedizin, Arbeitsschutz und Ergonomie. 2020: 1–4.

[14] Maurer-Grubinger C, Holzgreve F, Fraeulin L, Betz W, Erbe C, Brueggmann D, Wanke EM, Nienhaus A, Groneberg DA, Ohlendorf D (2021). Combining Ergonomic Risk Assessment (Rula) with Inertial Motion Capture Technology in Dentistry—Using the Benefits from Two Worlds. Sensors..

[15] Ahmadi Salim, Klingelhöfer Doris, Erbe Christina, Holzgreve Fabian, Groneberg David A (2021). Oral Health: Global Research Performance under Changing Regional Health Burdens. Int J Environ Res Public Health.

[16] Blume Kim, Sarah Fabian, Holzgreve Laura, Fraeulin Christina, Erbe Werner, Betz, Eileen M, Wanke Doerthe, Brueggmann Albert, Nienhaus (2021). Christian Maurer-Grubinger, and David A Groneberg. “Ergonomic Risk Assessment of Dental Students—Rula Applied to Objective Kinematic Data. Int J Environ Res Public Health.

[17] Holzgreve F, Fraeulin L, Betz W, Erbe C, Wanke EM, Brüggmann D, Nienhaus A, Groneberg DA, Maurer-Grubinger C, Ohlendorf D (2022). A Rula-Based Comparison of the Ergonomic Risk of Typical Working Procedures for Dentists and Dental Assistants of General Dentistry, Endodontology, Oral and Maxillofacial Surgery, and Orthodontics. Sensors..

[18] Unfallversicherung, Deutsche Gesetzliche. “Dguv Information 215–410.“ Bildschirm-und Büroarbeitsplätze Leitfaden für die Gestaltung (2019).

[19] Morfeld M, Kirchberger I, Bullinger M. Sf-36 Fragebogen Zum Gesundheitszustand: Deutsche Version Des Short Form-36 Health Survey, 2011.

[20] Blair Stephanie, Duthie Grant, Robertson Sam, Hopkins William, Ball Kevin (2018). "Concurrent Validation of an Inertial Measurement System to Quantify Kicking Biomechanics in Four Football Codes. J Biomechan.

[21] Robert-Lachaine X, Mecheri H, Larue C, Plamondon A (2017). Validation of Inertial Measurement Units with an Optoelectronic System for Whole-Body Motion Analysis. Med Biol Eng Comput.

[22] Teufl W, Miezal M, Taetz B, Fröhlich M, Bleser G (2018). Validity, Test-Retest Reliability and Long-Term Stability of Magnetometer Free Inertial Sensor Based 3d Joint Kinematics. Sensors..

[23] McAtamney L, Nigel Corlett E. Rula: A Survey Method for the Investigation of Work-Related Upper Limb Disorders. Appl Ergon. 1993;24(2):91–9. 10.1016/0003-6870(93)90080-s 15676903

[24] Middlesworth M. Rula: A Step-by-Step Guide. Ergonomics Plus Inc., https://ergo-plus.com/wp-content/uploads/RULA-A-Step-by-Step-Guide1.pdf (Accessed 05.06.2021).

[25] Hoehne-Hückstädt U, Herda C, Ellegast R, Hermanns I, Hamburger R, Ditchen D (2007). Bgia Report 2/2007.

[26] Vignais N, Bernard F, Touvenot G, Sagot JC (2017). Physical Risk Factors Identification Based on Body Sensor Network Combined to Videotaping. Appl Ergon.

[27] Bazazan A, Dianat I, Feizollahi N, Mombeini Z, Shirazi AM, Castellucci HI (2019). Effect of a Posture Correction-Based Intervention on Musculoskeletal Symptoms and Fatigue among Control Room Operators. Appl Ergon..

[28] Govil Nandini, DeMayo William M, Hirsch Barry E, Andrew AMcCall (2017). Optimizing Positioning for in-Office Otology Procedures. Otolaryngol–Head Neck Surg.

[29] Rodrigues MS, Leite RD, Lelis CM, Chaves TC (2017). Differences in Ergonomic and Workstation Factors between Computer Office Workers with and without Reported Musculoskeletal Pain. Work..

[30] Taieb-Maimon M, Cwikel J, Shapira B, Orenstein I (2012). The Effectiveness of a Training Method Using Self-Modeling Webcam Photos for Reducing Musculoskeletal Risk among Office Workers Using Computers. Appl Ergon..

[31] Ertekin Ersen, Özge EGünaydın (2021). Neck Pain in Rounded Shoulder Posture: Clinico-Radiologic Correlation by Shear Wave Elastography. Int J Clin Pract.

[32] Goodman G, Landis J, George C, McGuire S, Shorter C, Sieminski M, Wilson T (2005). Effectiveness of Computer Ergonomics Interventions for an Engineering Company: A Program Evaluation. Work..

[33] Mohammadipour Fariborz, Pourranjbar Mohammad, Naderi Sasan (2018). "Work-Related Musculoskeletal Disorders in Iranian Office Workers: Prevalence and Risk Factors. J Med Life.

